# Association between visual impairment and risk of suicide: Protocol for a systematic review and meta-analysis

**DOI:** 10.1371/journal.pone.0284355

**Published:** 2023-04-12

**Authors:** Chung Young Kim, Ahnul Ha, Sung Ryul Shim, In Boem Chang, Young Kook Kim

**Affiliations:** 1 Department of Ophthalmology, Seogwipo Medical Center, Seogwipo-si, Korea; 2 Seoul National University College of Medicine, Seoul, Korea; 3 Department of Ophthalmology, Jeju National University Hospital, Jeju-si, Korea; 4 Department of Ophthalmology, Jeju National University College of Medicine, Jeju-si, Korea; 5 Department of Health and Medical Informatics, Kyungnam University College of Health Sciences, Changwon, Korea; 6 Seoul On Eye Clinic, Seoul, Korea; 7 Seoul National University Hospital, Seoul, Korea; Universidade Federal do Rio Grande do Sul, BRAZIL

## Abstract

**Introduction:**

Suicide is an important public health problem. Well-established risk factors of suicide include depression, family history of mental disorders, substance problem, chronic physical illness, and others. Sensory impairment, especially visual impairment (VI), has a critical impact on both mental and physical health. However, the association between VI and risk of suicide has not been thoroughly investigated and remains controversial. Our aim is to systematically review and meta-analyze the current evidence on the association between VI and risk of suicide and to evaluate the direction and magnitude of the association.

**Methods and analysis:**

We aim to search PubMed, EMBASE and the Cochrane Library to identify all population-based studies on the association between VI and risk of suicide. Two reviewers will independently conduct study selection, data extraction and risk of bias (ROB) assessment. The Newcastle-Ottawa scale will be applied to evaluate the methodologic quality of the included studies for ROB assessment. The primary outcome measure will be the relative risk (RR) of suicide, and the secondary outcome measures will be the risks of suicidal ideation (SI) and suicide attempt (SA). Estimates of risk with 95% confidence intervals (CIs) for suicide, SI and SA, respectively, will be calculated and summarized. We will perform random-effects meta-analyses to combine the pooled effects. Meta-regression will be applied to investigate the effects of multiple factors across studies. Subgroup and sensitivity analyses will be conducted for screening of any potential sources of heterogeneity. Publication bias will be evaluated by funnel plot and Begg and Mazumdar correlation testing. The body of evidence will be assessed using the Grading of Recommendations, Assessment, Development and Evaluation (GRADE) approach.

**Conclusion:**

This article presents a study protocol for investigating the association between VI and risk of suicide. The findings of this study will contribute to our current knowledge of the impact of VI as a risk factor of suicide. In addition, meta-regression and subgroup analyses will provide further insights to factors affecting the association between VI and suicide risk.

**Trial registration:**

**Systematic review registration:** PROSPERO CRD 42022325106.

## Introduction

Suicide is both a critical public health problem and preventable. Each year, over 800,000 people die by suicide, which is among the leading causes of death worldwide [1]. Suicide is the act of intentionally ending one’s own life. Suicidal behavior (SB) includes both completed suicide and suicide attempt (SA), a nonfatal self-directed injurious act committed with the intent to die [2, 3]. Suicidal ideation (SI) is defined as either passive thoughts about wanting to be dead or active thoughts about killing oneself, neither of which is accompanied by preparatory behavior [4]. The concept of suicide includes a spectrum of conditions from SI to SB, which usually present simultaneously. To date, several risk factors for SI and SB have been reported. Mental illness (especially depression and other mood disorders), family history of suicide or mental disorders, substance abuse, chronic illness and disability, specific population status (e.g., migrant, prisoner, non-heterosexual), social isolation or access to lethal means increase the risk of suicide [5–9]. In older populations, sleep disorder, decreased mobility, poor quality of life and serious functional impairment have been reported to be related with the increased risk [10–14].

Visual impairment (VI) is estimated to affect between 9 and 18% of older adults and is a growing public health problem due to the demographic shift such populations [15, 16]. It can lead to higher risk of suicide via factors such as cognitive decline, limited mobility, disability, difficulties in social participation, and poor self-related health [17–20]. However, the statistical significance of VI as a risk factor of suicide remains controversial. In some studies, VI has been found to be a risk factor in combination with depression or poor general health [21–23]. However, in other studies, VI has not shown significant hazard ratios or strong correlations with increased risk of suicide [24–26].

The global burden of suicide and its impact on public health is immense, and it is imperative to identify risk factors, especially modifiable ones such as VI. Therefore, meta-analyses evaluating the directionality and magnitude of any association between VI and risk of suicide are required. Herein, we present a protocol for systematic review of the current evidence on the association between VI and risk of suicide. Based on this protocol, a meta-analysis of published studies will be conducted to determine whether VI in fact escalates the risk of suicide.

## Methods and analysis

This protocol adheres to the Preferred Reporting Items for Systematic Reviews and Meta-Analyses protocol (PRISMA-P) guidelines [27]. The protocol was registered in the PROSPERO database (CRD 42022325106).

### Eligibility criteria

In this systematic review and meta-analysis, we will include studies satisfying the following criteria: (1) population based, (2) VI reported as covariate, (3) suicide, SI and SA included as outcome measures, (4) Odds ratios (ORs) or relative risks (RRs) with 95% confidence intervals (CIs) as measure of association, or allowance of calculation from count data provided in the article. We will exclude (1) studies not conducted with human subjects, (2) narrative and/or systematic review, case reports, commentaries, editorials, or conference abstracts, and (3) reports lacking a clear definition of visual impairment or a clear description of suicide assessment.

### Search strategy

With the assistance of a medical librarian, we will search for published studies in the following databases: PubMed (MEDLINE), EMBASE and the Cochrane Library. We will perform the literature search using the following medical subject headings (MeSH) and text words related to visual impairment and suicide: *visual impairment; blindness; suicide; suicidal ideation; suicide attempt; association*. No restrictions regarding study design, date or language will be imposed. For the reference lists of prospectively identified systematic reviews and meta-analyses, we will perform manual searches to identify potentially relevant but missed reports by the electronic searches. The full search strategies will be provided in online supplemental file.

### Study selection

**Fig 1:**
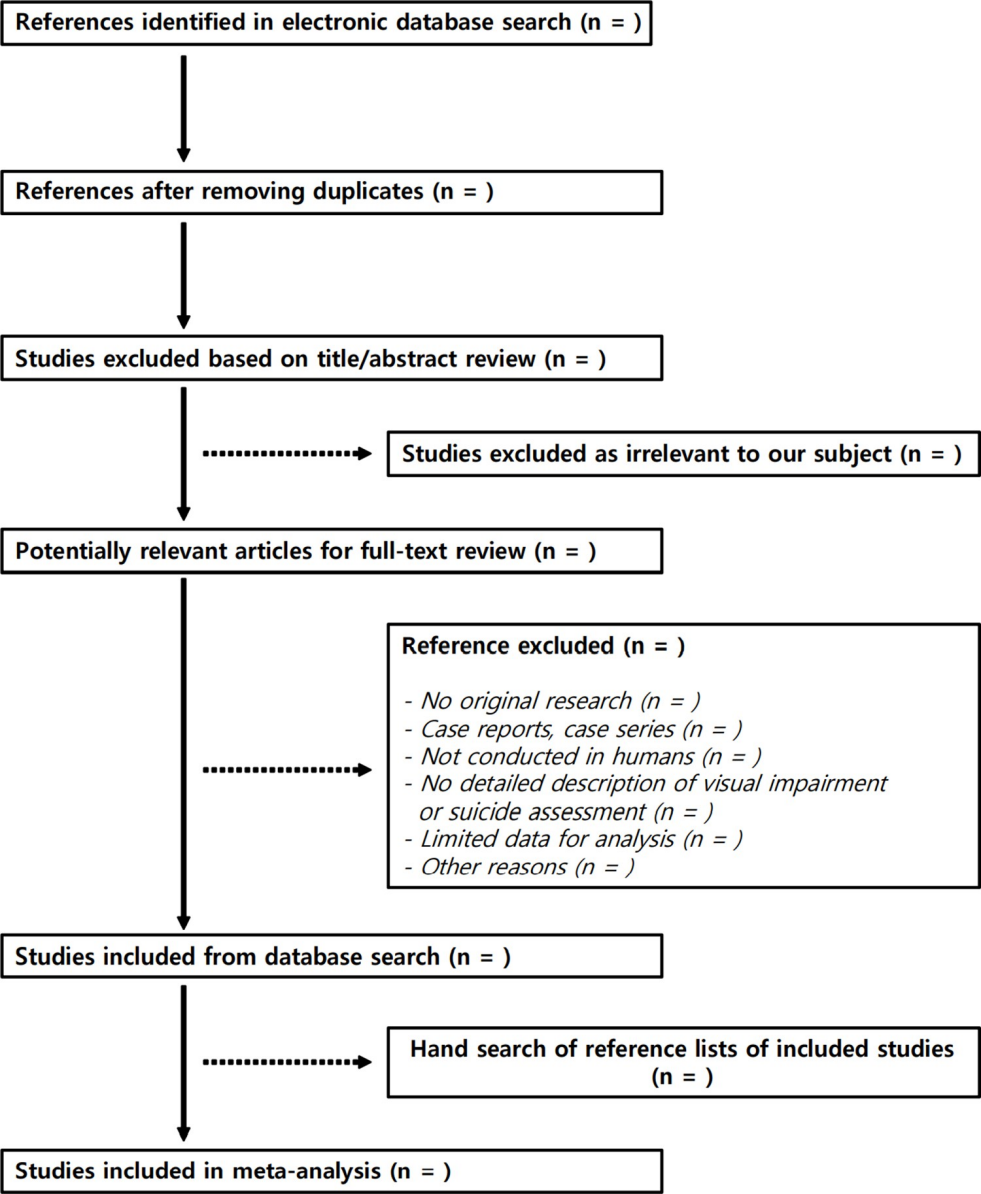
Preferred reporting items for systematic reviews and meta-analyses flow diagram of the study selection process.

Titles and abstracts will be screened by two reviewers independently to identify potentially eligible studies based on the inclusion criteria. Then for each identified study, the reviewers will evaluate the full-text papers for inclusion. In either of these two stages, any disagreements will be arbitrated by discussion or consultation with a third investigator. The inter-reviewer agreements will be evaluated in terms of Cohen’s kappa coefficient (*κ*). When there are multiple publications are present for the identical study population, only the most comprehensive and latest report with the largest sample size will be included (after duplicate verification). The stepwise study selection process will be summarized in a PRISMA flow diagram (Fig 1).

### Data extraction

For each included study, the two reviewers will independently extract data using a standardized data collection form. Study characteristics of interest will include (1) study ID (name of the first author, publication year), (2) baseline study year, (3) country of study, (4) ethnicity of subjects, (5) language, (6) number of subjects, (7) characteristic of subjects, (8) ages and sexes of subjects, (9) measures of association (RRs, ORs) with 95% CIs, and (10) confounding factors adjusted.

In terms of VI, (1) the definition of VI and (2) the method of VI assessment will be extracted. For suicide, (1) the definition of SI and SA will be extracted, as will (2) the confirmation method of suicidal death. If necessary, we will contact the corresponding author of any study to request specific information not presented in the article.

### Risk of bias (ROB) assessment

To assess the methodologic quality of included studies, we will apply the Newcastle-Ottawa Scale, which is a validated tool for assessment of quantitative cross-sectional, case-control and cohort studies [28]. Risk of bias including selection, comparability, exposure/outcome, or any other type of bias will be determined by additional investigation of included studies.

### Outcomes

The primary outcome measure will be the RR of suicide, and the secondary outcome measures will be the risks of SI and SA.

### Data synthesis and analysis

We will pool unadjusted and adjusted estimates of increased risk along with the corresponding 95% CIs for suicide, SI, and SA. When there are both unadjusted and adjusted estimates are presented, adjusted estimates will be included for analysis. We will calculate unadjusted measures of association when only count data are provided. If neither count data nor estimates of risk are available, we will calculate the standardized mortality ratio (SMR) based on the corresponding study’s reference-population statistics [29]. For studies reporting only subdivided RRs according to different severities of VI, we will synthesize those measures to acquire an overall estimate for any severity of VI. If individual studies present different ratio measures of association (e.g., rate ratio, OR, RR), we will regard those estimates as fairly similar, since the occurrence of suicide would be rare. A random-effects model will be applied to synthesize study-specific measures of association. Risk of suicide, SI and SA will be analyzed separately. We will calculate pooled estimates presented as ORs with 95% CIs and will provide them in the forest-plot format. All of the 95% CI and P values will be 2-sided, and P < 0.05 will be considered to represent statistical significance. All of the statistical analyses will be performed with R 4.0.4 software (The R Foundation for Statistical Computing).

### Subgroup and sensitivity analyses

We will perform a subgroup analysis to evaluate whether there are any potential sources of heterogeneity. This analysis will be performed according to the geological continent of the study conducted and the methodology of VI assessment (objective visual acuity measurement or answer to survey questionnaire). In order to account for potential heterogeneity and assess the robustness of the results, a sensitivity analysis will be conducted. To that end, the analyzes for the primary outcome will be repeated after excluding studies with possible outliers. This sensitivity analysis will offer a comprehensive perspective on the findings of the study.

### Grading of evidence

The body of evidence will be assessed using the Grading of Recommendations, Assessment, Development and Evaluation (GRADE) approach [30], which will entail two independent researchers’ grading of the evidence based on the following considerations: risk of bias, heterogeneity, indirectness, and publication bias.

### Meta-regression

Meta-regression analyses will be performed to investigate the possible causes for different effect sizes across studies. To explain substantial heterogeneity in the outcome of interest across studies, differences in characteristics of the included studies or study population will be investigated. This will enable to evaluate the particular influence of multiple factors that possibly contribute to the impact of VI on risk of suicide.

### Publication-bias assessment

Publication bias will be evaluated (1) qualitatively by funnel plot and (2) quantitatively by Begg and Mazumdar correlation testing (a statistical analog of the funnel plot). The Begg and Mazumdar test will assess whether there is any significant correlation between the effect estimates and their variances. If there is no such correlation, it will suggest that study selection process was conducted in an unbiased way.

### Heterogeneity assessment

We will assess clinical and methodological heterogeneity by examining the important clinical characteristics and methodological differences. Statistical heterogeneity will be assessed using the *I*^*2*^ index, which represents the between-study variation percentage attributable to heterogeneity (not to sampling error). Values of approximately 25, 50, and 75% will represent low, medium, and high heterogeneity, respectively.

### Patient and public involvement

Patients or members of the public will not be involved in the design, conduct, reporting, or dissemination plans of this study. Only data already existent in the literature will be included in this study.

### Ethics and dissemination

Ethics approval will not be required for the current study, because this will be a review that synthesizes only previously reported data. The findings will be shared with the public by either academic presentation at a conference or peer-reviewed publication. Additionally, significant protocol adjustments will be documented and updated on PROSPERO. The date(s) of adjustments and a summary of changes will be presented as a supplement.

## Discussion

VI has been reported to be related with increased risk of suicide, however, statistical significance remains controversial. This study will aim to identify the directionality and magnitude of any association between VI and risk of suicide by systematic review, meta-analysis and meta-regression.

The strengths of our study is that this will be a comprehensive systematic review, meta-analysis and meta-regression analysis investigating the association between VI and risk of suicide. Second, the current study will strictly adhere to both the Meta-analysis Of Observational Studies in Epidemiology and the Preferred Reporting Items for Systematic Reviews and Meta-Analyses guidelines [31, 32]. Third, this study will undertake a rigorous selection process and ROB assessment for each study, to be performed by two independent authors and arbitrated by a third investigator. Fourth, our study will not only investigate the strength of association but also reveal the factors affecting the association between VI and suicide risk.

We anticipate limitations in the heterogeneity of published data and a wide variance of definition, assessment of VI and suicide across studies. We aim to overcome this by extracting or requesting raw data in each study if available and investigating statistical methodology to pool them together.

In summary, this article presents a study protocol for investigating the association between VI and risk of suicide. The findings of this study will contribute to our current knowledge of the impact of VI as a risk factor of suicide. In addition, meta-regression and subgroup analyses will provide further insights to sociodemographic and clinical factors affecting the association between VI and suicide risk.

## Supporting information

S1 File PRISMA-P checklist. (DOCX)

S2 File Search terms. (PDF)

## Abbreviations and acronyms

VI: visual impairment
SB: suicidal behavior
SI: suicidal ideation
SA: suicide attempt
OR: odds ratio
RR: relative risk
CI: confidence interval
ROB: risk of bias
SMR: standardized mortality ratio
