# Supplementary material for: Association between visual impairment and risk of suicide: Protocol for a systematic review and meta-analysis
Source: PLoS One. 2023 Apr 12;18(4):e0284355. doi: 10.1371/journal.pone.0284355 (PMC10096229; doi:10.1371/journal.pone.0284355)
Supplement: S2 File — (PDF) [file pone.0284355.s002.pdf]

Search Terms

Pubmed (MEDLINE)

(“Vision, low”[Mesh] OR “Low vision”[Tiab] OR “Blindness”[Mesh] OR “Blind*”[Tiab] OR “Visually impaired persons”[Mesh] OR “Sensory”[Tiab] OR “Visual acuity”[Mesh] OR “Visual”[Tiab] OR “Impairment”[Tiab] OR “Disability”[Tiab]) **AND (**“Suicide”[Mesh] OR “Suicid*”[Tiab] OR “Self-injurious behavior”[Mesh] OR “Self mutilation”[Mesh] OR “Drug overdose”[Mesh])

Cochrane Central Register of Controlled Trials (CENTRAL) in The Cochrane Library (Wiley)

#1 MeSH descriptor Vision, Low
#2 MeSH descriptor Blindness
#3 MeSH descriptor Visually Impaired Persons
#4 MeSH descriptor Visual Acuity
#5 “Low vision” OR “Blind*” OR “Sensory” OR “Visual” OR “Impairment” OR “Disability”
#6 (#1 OR #2 OR #3 OR #4 OR #5)
#7 MeSH descriptor Suicide
#8 MeSH descriptor Self-injurious behavior
#9 MeSH descriptor Self mutilation
#10 MeSH descriptor Drug overdose
#11 “Suicid*”
#12 (#7 OR #8 OR #9 OR #10 OR #11)
#13 (#6 AND #12)

EMBASE (Ovid)

(‘Low vision’/exp OR Blindness/exp OR ‘Visually Impaired Persons’/exp OR ‘Visual Acuity’/exp OR Blind* OR “Sensory” OR “Visual” OR “Impairment” OR “Disability”) AND (Suicide/exp OR Automutilation/exp OR ‘Drug overdose’/exp OR Suicid*)
